# Health Care Waste Segregation Behavior among Health Workers in Uganda: An Application of the Theory of Planned Behavior

**DOI:** 10.1155/2016/8132306

**Published:** 2016-12-27

**Authors:** Martha Akulume, Suzanne N. Kiwanuka

**Affiliations:** School of Public Health, Makerere University, P.O. Box 7072, Kampala, Uganda

## Abstract

*Objective*. The goal of this study was to assess the appropriateness of the theory of planned behavior in predicting health care waste segregation behaviors and to examine the factors that influence waste segregation behaviors.* Methodology*. One hundred and sixty-three health workers completed a self-administered questionnaire in a cross-sectional survey that examined the theory of planned behavior constructs (attitudes, subjective norms, perceived behavioral control, and intention) and external variables (sociodemographic factors, personal characteristics, organizational characteristics, professional characteristics, and moral obligation).* Results*. For their most recent client 21.5% of the health workers reported that they most definitely segregated health care waste while 5.5% did not segregate. All the theory of planned behavior constructs were significant predictors of health workers' segregation behavior, but intention emerged as the strongest and most significant (*r* = 0.524, *P* < 0.001). The theory of planned behavior model explained 52.5% of the variance in health workers' segregation behavior. When external variables were added, the new model explained 66.7% of the variance in behavior.* Conclusion*. Generally, health workers' health care waste segregation behavior was high. The theory of planned behavior significantly predicted health workers' health care waste segregation behaviors.

## 1. Introduction

Health care waste (HCW) is a byproduct of health care activities and is comprised of materials ranging from used needles and syringes to soiled dressings, body parts, diagnostic samples, blood, chemicals, pharmaceuticals, medical devices, and radioactive materials [1]. The Ugandan Ministry of Health [2] classifies this waste into categories which include the following:

- Highly infectious waste, for example, amputated limbs, placenta, extracted teeth, used test tubes and test kits, used blood bags, and all food items from highly infectious patients.
- Infectious waste including used gauze, used cotton, pad and cloths, and contaminated bottles for infusion fluids.
- Sharps: used syringes, needles' cut-off infusion sets, used scalpels, broken glass, ampoules, and cannulas.
- Pharmaceutical waste: expired and damaged drugs, lab reagents, empty vials, and heavy metals.
- Nontoxic waste: food items, empty bottles for drinks, paper, and packaging material.

The management of HCW requires intense devotion and diligence because, if poorly managed, it may pose risk to health care workers, waste handlers, patients, and the entire community [1]. The key to effective management of HCW is segregation at point of generation [3]. Segregation means placing the various categories of waste into different color coded bins with liners. According to the Ministry of Health, Uganda [2], the recommended color coding scheme is green bin with liner for noninfectious plastics, black bin with liner for other noninfectious wastes, yellow safety box for sharps, yellow bin with liner for infectious waste, red bin with liner for highly infectious waste, and brown bin with liner for pharmaceutical waste.

Despite the necessity of segregation in Health Care Waste Management (HCWM), some countries either lack proper rules and regulations on HCW segregation or do not impose them; hence the Health Care Waste Management Systems (HCWMS) are insufficient [4]. An assessment carried out in 22 developing countries in 2002 showed that 18% to 64% of the Health Care Facilities (HCF) did not employ appropriate waste disposal methods [5].

In Uganda, waste generated in hospital averages 92 Kg per day and 42 Kg per day at Health Center (HC) IV level while HC III and HC II levels generate 25 and 20 Kg of waste, respectively, per day [6]. The Ugandan Ministry of Health developed a policy on injection and HCWM [6]. However, the HCFs in Uganda including those in Pallisa district have insufficient HCWMS and there is strong evidence that HCW is not segregated [7]. Moreover, Pallisa district lacks proper HCWM facilities [8], thus posing a risk for insufficient HCW segregation. Based on the “polluter pays principle,” the responsibility of managing waste lies upon the waste producer (health worker). This means that HCW segregation is the duty of the health worker and depends heavily on health worker's behavior.

Azjen's theory of planned behavior (TPB) has been used to predict behavior and understand its causes [9]. It posits that a person's behavior is highly determined by his or her intention (readiness), which is considered the most immediate determinant of behavior [10]. Intention in turn depends on a person's attitudes (feeling of favorableness or unfavorableness) towards the behavior, influence of subjective norms (perceived social pressure), and perceived behavioral control (a person's perception of his or her ability to perform a behavior). Perceived behavioral control not only affects behavior through intention but may also influence it directly [10]. The TPB was proposed in 1985 by Ajzen through his article “From Intentions to Actions: A Theory of Planned Behavior” as an expansion of the Theory of Reasoned Action [11]. The Theory of Reasoned Action (TRA) explained that if people had positive attitudes and if they thought that their significant others wanted them to perform a specific behavior, that would result in higher intentions and they would be very likely to perform that behavior [12]. However, this theory had some limitations since behavior is not solely determined by intention where an individual's control over the behavior is incomplete. As a result, Ajzen [11] introduced the TPB by adding a new construct, “perceived behavioral control.” Perceived behavioral control comprises of the internal and external resources that affect behavior either directly or indirectly through intentions [13], for example, presence of color coded bins.

According to Ajzen [10], the TPB is, in principle, open to the addition of other predictors as long as their inclusion is justified theoretically and they capture significant variance in behavior or behavioral intention. For instance, Ajzen recommended that, in some contexts, one needs to not only consider the influence of subjective norms but also moral obligation. Moral obligation is defined as an individual's perception of moral correctness or incorrectness of undertaking a certain behavior. Furthermore, a study by Ann [14] demonstrated the necessity for cautious collection of basic demographic data during the data collection process, as this data may possibly offer hints to the significance of the TPB in different research contexts encompassing various individuals.

The TPB has been used successfully in different fields to study behavior with particular emphasis on solid waste management behaviors and other health related behaviors. For example, a study on teacher candidates' recycling behaviors revealed that teacher candidates possessing more favorable attitudes and subjective norms together with a greater perceived behavioral control tended to have stronger intention to engage in a recycling behavior and stronger intention could result in more active commitment to recycling behavior [15]; a study on household waste behaviors among a community sample in Iran revealed that attitude, subjective norms, perceived behavioral control, moral obligation, and intention significantly predicted household behavior [16]; using the theory of planned behavior to determine recycling and waste management behaviors in Bristol city showed that perceived behavioral control followed by attitude was significant predictor of intention to recycle [17].

Despite the support for the TPB in explaining human behavior (specifically waste management behaviors), not much has been studied about HCW segregation in the context of the TPB in Pallisa district and elsewhere. However many studies have been carried out to assess the health workers' awareness/knowledge, attitude, and practices towards HCW segregation [18–20]. Because these studies use various approaches, they have got a number of hypothetical weaknesses. They do not examine the relationship between health workers' readiness to segregate health care waste (intentions) and health workers' perception of their ability to segregate HCW (perceived behavioral control) with behavior. As a result, they provide inadequate information on the factors associated with health worker's HCW segregation behaviors.

Yet, with the current rate of population growth, in the future there will be a large increase in HCW generation due to the high number of patients [21]. This will constitute a big problem to HCWM which is mostly at the mercy of the health workers' HCW segregation behaviors.

The present study therefore used Ajzen's TPB as theoretical framework to systematically examine the factors associated with health workers' HCW segregation behaviors. Not only has this theory been proven to be one of the most powerful, influential, and predictive model for explaining human behaviors [22–25], but also it provides a useful guide for designing intervention strategies to change or maintain behavior.

## 2. Materials and Methods

The HCFs in Pallisa district are grouped into different levels from HC II, HC III, and HC IV to general hospital. Different facility levels provide different services, with the number of services increasing with the level. At HC II level, only outpatient services and community outreaches are provided while HC IIIs provide basic preventive, promotive and curative care, laboratory services for diagnosis, and maternity care. In addition to the services provided by HC IIIs, HC IV s also provide minor surgeries, blood transfusion services, and emergency obstetric care while general hospitals provide preventive, promotive, curative maternity, inpatient health services, surgery, blood transfusion, laboratory, and medical imaging services.

A cross-sectional study was carried out with a quantitative method of data collection. The target population consisted of health workers working in either private or governmental HCFs in Pallisa district, and these were from the HC II level to general hospital. Two hundred and twelve questionnaires were distributed to the health workers in the various HCFs and one hundred sixty-three were returned, yielding to a response rate of 76.9%.

### 2.1. Sampling and Procedure

Census sampling was used for the survey and all available health workers were invited to participate in the study. The inclusion criteria were health workers who directly dealt with patients and generated waste as a result of their interactions. Questionnaires were distributed to health workers during the mass immunization training that was carried out at the beginning of the year. The health workers who did not attend the training were approached at the various HCFs. Due to different working shifts, some health workers were not present at the HCFs. To ensure that these health workers got information about the study, extra questionnaires were left with the various officials in charge of the HCFs or representatives at the HCFs. Consent forms and information sheets clarifying the purpose of the study were attached to the questionnaires.

### 2.2. Questionnaire

Following the TPB guidelines [26], a self-administered questionnaire was designed by the author to assess health workers' HCW segregation behaviors, TPB constructs, and external factors influencing health workers' waste segregation behaviors. The questionnaire was prepared in English and consisted of 53 questions with the majority being closed-end questions.

#### 2.2.1. Behavior

To assess health workers' HCW segregation behavior, 2 items, for example, “I segregated health care waste for the last client I attended to” were used. These were measured on a 5-point Likert scale with points ranging from 1 (most definitely) to 5 (not at all). Cronbach's alpha for this scale was 0.732.

#### 2.2.2. Intention

Two items were used to assess the behavioral intention of the health workers, for example, “at my work place, I intend to segregate health care waste over the next month.” Responses were measured on a 5-point Likert scale ranging from 1 (unlikely) to 5 (likely). Cronbach's alpha for this scale was 0.741.

#### 2.2.3. Perceived Behavioral Control

Five items were included to capture perceived behavioral control over HCW segregation, for example, “I am capable of segregating health care waste.” This was measured on a 5-point scale with points from 1(strongly disagree) to 5 (strongly agree). Cronbach's alpha for this scale was 0.749.

#### 2.2.4. Subjective Norms

Subjective norms were captured with 8 items such as “most people who are important to me think I should segregate HCW.” Each item was rated on a 5-point scale from 1 (strongly agree) to 5 (strongly disagree). Cronbach's alpha for this scale was 0.831.

#### 2.2.5. Attitudes

Attitudes were assessed with 12 items such as “segregating HCW is useful.” Responses were measured on a 5-point Likert scale ranging from 1 (strongly disagree) to 5 (strongly agree). Cronbach's alpha for this scale was 0.853.

#### 2.2.6. Moral Obligation

As recommended by Ajzen [10], 2 items were included to assess moral obligation such as “it is moral to segregate health care waste” on a five-point Likert scale of 1 (strongly disagree) to 5 (strongly agree). Cronbach's alpha for this scale was 0.677.

#### 2.2.7. External Variables

In addition to the TPB constructs, respondents' social demographic, personal, organizational, and professional characteristics were assessed.

### 2.3. Pretesting

The original version of the questionnaire, which was prepared in English, was pilot-tested on a sample of ten nursing students at Mulago Hospital and these were not from the study area. Pretesting was done to assess whether the questions and statements were understood by the respondents the same way the investigators had intended. Questions and statements that were not clear to the respondents were reviewed in the final version of the questionnaire.

### 2.4. Data Analysis

The data analysis was conducted in several steps. First, the distributions of each variable were inspected to check for errors. Reliability tests were then performed for each TPB construct using Cronbach's alpha in SPSS.

The Likert scales for each TPB construct (behavior, intention, PBC, attitude, and subjective norms) were computed. The items were dichotomized into “yes” and “no.” Positive items were dichotomized separately from the negative items. The dichotomized items were then recoded in SPSS with yes = 1 and no = 0. To obtain the final measure of each variable, the sum of the recoded items was obtained. The sum was further recoded and dichotomized into “yes” and “no.” Depending on the number of items measuring each variable, low values of sum indicated no while high values of the sum indicated yes.

Descriptive statistics were used to investigate participants' characteristics. Beyond descriptive statistics, associations were analyzed using correlations and regressions.

### 2.5. Ethical Considerations

Ethical clearance was obtained from the School of Public Health, Makerere University, and permission was obtained from the relevant district authorities. The respondents were briefed about the goals of the study. They were also informed about the study and their rights to refuse to join or to decline to answer any question they felt uncomfortable with. The participation in the study was voluntary and respondents were required to fill in consent forms before they participated in the study. The participants were also given an option of contacting the researcher in case they wanted more information.

To ensure confidentiality, no names or any identifying information was collected from the respondents.

## 3. Results

### 3.1. Participants' Characteristics

As shown in Table 1, slightly more than half of the respondents were male (52.1%). Regarding their professional characteristics, 50.9% were nurses/midwives and approximately 76% did not have knowledge on color coded bins and 60.7% had not had adequate training on HCW segregation. See Table 1.

### 3.2. Health Workers' Health Care Waste Segregation Behavior

**Figure 1:**
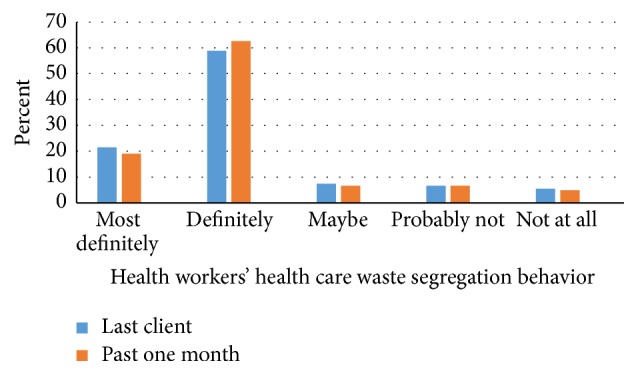
Health workers' HCW segregation behaviors in the past one month and for the last client attended to.

Nineteen percent health workers reported most definitely segregating their HCW, while 62.2% definitely segregated HCW, 6.7% were not sure if they segregated HCW, 6.7% probably did not segregate HCW, and 4.9% did not segregate in the past one month. Moreover 21.5% most definitely segregated HCW, 58.9% definitely segregated HCW, 7.4% were not sure if they segregated HCW, 6.7% probably did not segregate HCW, and 5.5% did not segregate for their last client (Figure 1).

### 3.3. Factors Associated with Actual Health Workers' HCW Segregation Behaviors

Pearson's correlation coefficients were computed for the TPB constructs and external variables. As shown in Table 2(a), significant correlations were found between health workers' HCW segregation behaviors and the TPB constructs. As anticipated, all the TPB constructs were significantly correlated with intention and behavior, with intention emerging as the strongest behavioral correlate (*r* = 0.524, *P* value < 0.01). The matrix also revealed a moderate correlation (*r* = 0.452, *P* value < 0.01) between perceived control and behavior. Attitudes, subjective norms, and moral obligation had low correlations (*r* = 0.293, *P* value < 0.01), (*r* = 0.377, *P* value < 0.01), and (*r* = 0.188, *P* value < 0.01), respectively, with behavior. Generally, correlations between the TPB constructs and behavior were stronger than those between the TPB constructs and intention implying that the TPB constructs were likely to influence behaviors directly than through interactions (intention).

Regarding external variables, only ownership (*r* = −0.210, *P* value < 0.01), level of the HCF (*r* = 0.208, *P* value < 0.01), knowledge on HCW segregation (*r* = −0.299, *P* value < 0.01) and color coded bins (*r* = 0.209, *P* value < 0.01), perceived risk (*r* = −0.228, *P* value < 0.01), and adequate training (*r* = −0.176, *P* value < 0.05) had significant correlation with behavior. Whereas level of HCF was positively associated with behavior, knowledge on HCW and color coded bins, perceived risk, and adequate training had negative correlation with behavior (Table 2(b)).

### 3.4. Significance of the TPB in Predicting HCW Segregation Behavior

As shown in Table 3, the TPB constructs (attitudes, subjective norms, perceived control, and intention) explained 52.5% variance in health workers' HCW segregation behaviors. Intention (OR 18.1, *P* value < 0.001) and perceived control (Odds Ratio 9.61, *P* value < 0.001) were the biggest predictors of HCW segregation behaviors. See Table 3.

The Hosmer and Lemeshow goodness-of-fit test was not significant implying that the TPB was a robust model for predicting health workers' HCW segregation behaviors.

### 3.5. Significance of External Variables and the TPB Constructs in Predicting Health Workers' HCW Segregation Behavior

As shown in Table 4, a multivariable analysis adjusting for both TPB constructs and external variables with *P* values < 0.1 revealed that 66.7% variance in health workers' HCW segregation behavior was explained by the new model (see Table 4).

The multivariable analysis also revealed that attitude, subjective norms, moral obligation, ownership of facility, level of the HCF, knowledge on HCW segregation, perceived risk, and adequate training lost significance in predicting health workers' HCW segregation behavior.

## 4. Discussion

Understanding the factors underlying health workers' HCW segregation behaviors is a vital step towards developing interventions to improve HCW segregation. Most health workers reported that they segregated HCW (most definitely and definitely) in the past one month and for the last client. This is in contrast with the results of earlier studies which revealed that the HCFs in Uganda have insufficient HCWMS and there is strong evidence that HCW is not segregated [7]. According to Muhwezi et al. [27], there were lower levels of waste segregation with different categories of HCW being mixed up within the color coded bins. This inconsistency could have resulted due to the methods of data collection used. Whereas Muhwezi et al. [27] used observation, interviews, and participatory methods, this study only employed self-reports by the health workers which were not verified by direct observation.

### 4.1. Relationship between the TPB Constructs and the Segregation Behavior

Based on the results from correlation analyses, intention, attitudes, perceived behavioral control, moral obligation, and influence of subjective norms considerably predicted health workers' HCW segregation behavior and were significant with varying strength. Behavioral intention was nonetheless found to be the strongest behavioral correlate, meaning that indeed intention is the most immediate determinant of behavior as evidenced by other studies [22, 28]. On the contrary, one study by Fila and Smith [29] showed that no associations were found between intentions and healthy eating behaviors indicating that, perhaps, although intention may predict some behavior, it may not consistently predict others.

Perceived behavioral control had a moderate correlation (*r* = 0.452, *P* value < 0.01) with health workers' HCW segregation behaviors implying that HCW segregation was not generally perceived to be a difficult or inconvenient task. This is consistent with the findings from another waste management behavior study carried out in Iran [16] where the correlation coefficient between participants' behavior and perceived behavioral control was 0.48, *P* value < 0.01. However, a study among teacher candidates [15] reported a nonsignificant relationship between recycling behaviors and perceived behavioral control implying that perceived behavioral control is not always significantly associated with recycling behaviors. Again this inconsistency may have been a result of the different methods used in data collection and analysis across studies. Subjective norms had a low correlation (*r* = 0.377, *P* value < 0.01) with health workers' HCW waste segregation behaviors.

Nevertheless, subjective norms were not the weakest determinant of health workers' HCW segregation behaviors. Attitudes (with a correlation coefficient of *r* = 0.293, *P* value < 0.01) had the weakest association with behavior among all the TPB constructs. This is inconsistent with the review of the TPB applicability to health related behaviors where subjective norms had less influence on behavior than attitudes [30]. The possible cause of this inconsistency could have been the difference in measurement of this construct: many authors use single-item measures [22] as opposed to the more dependable multi-item scale used in this study.

Moral obligation was found to be the weakest determinant of health workers' HCW segregation behaviors which is in contrast to the results of previous studies [16, 31] where moral obligation was found to be the strongest determinant of behavior. Ajzen [10] recommended the addition of moral obligation to some contexts so as to improve the predictive strength of the TPB. However moral obligation did not significantly improve the models' predictive strength in this study. This may imply that, for health workers, moral obligation is not a major influencing factor for segregation behavior and policy options should focus on other factors.

More support for the applicability of the TPB to health workers' HCW segregation behaviors is provided by the correlation of subjective norms, attitudes, and perceived behavioral control with intention. However, the correlation between the TPB constructs and intention was weaker than the correlation between behavior and TPB constructs. This could be an anomaly exclusive to health workers' HCW segregation behaviors, or it could even be related to the nature of the questionnaire (self-report).

### 4.2. The TPB as a Predictor of Health Workers' Actual HCW Segregation Behaviors

The TPB was found to be a useful model in predicting health workers' HCW segregation behaviors and accounted significantly for the variance (52.5%) in HCW segregation behaviors. This is consistent with the results from the study by Sutton [32] where the TPB explained between 19 and 38% variance in behavior. The high percentage of variance explained in this study could be attributed to the nature of the questionnaire (self-administered by the respondents). The regression analysis also showed that attitude and subjective norms lost their significance in predicting health workers' HCW segregation behaviors. This is not surprising since these were the weakest behavioral correlates (subjective norms; *r* = 0.377, *P* value < 0.01, and attitude; *r* = 0.293, *P* value < 0.01).

### 4.3. Influence of External Variables on Health Workers' HCW Segregation Behaviors

Including external variables as a separate construct may be redundant since perceived behavioral control (the internal and external resources that affect behavior either directly or indirectly through intentions) reflects external variables [33]. However, in this study, the role of external variables in predicting HCW segregation behavior was important to note since they tended to improve the predictability of the model because the variance in behavior explained by the model increased from 52.5% to 66.7%. The increase in variance of behavior implies that, in addition to the TPB constructs, external variables (sociodemographics and personal, professional, and organizational characteristics) are important in ensuring that health workers take up good segregation practices.

Only knowledge on HCW segregation and the use of color coded bins, perceived risk, adequate training, ownership, and level of facility had significant correlation with behavior. Level of HCF had a positive correlation with behavior (*r* = 0.208, *P* value < 0.01). This implies that health workers working in hospitals were more likely to segregate HCW than health workers working in HC IIs. This could be due to more frequent supervision in hospitals than in the health centers.

A negative correlation was found between HCW segregation behaviors and ownership of the HCF. This implies that health workers who worked in government HCFs were more likely to segregate HCW than those who worked in NGOs. This could be due to the support given to the former by government which makes them able to afford necessary resources required to segregate HCW. A surprising finding that knowledge on HCW segregation and the use of color coded bins was negatively correlated with HCW segregation behavior may be explained by the fact that Pallisa district lacks proper HCWM facilities [8] and therefore although health workers may be knowledgeable about HCW segregation, the absence of color coded bins in the HCFs restricts positive HCW behavior. Perceived risk and adequate training were negatively correlated with HCW segregation behaviors. This implies that health workers who perceived that poor HCW segregation caused risks to humans and those who had adequate training on HCW segregation were less likely to segregate HCW. This could be attributed to the lack of color coded bins in the HCFs. These findings make the importance of perceive behavioral control evident: the resources and opportunities available to a person to some extent dictate the likelihood of behavioral achievement (health care waste segregation) [10].

Surprisingly, most external factors (knowledge on HCW segregation and the use of color coded bins, perceived risk, and adequate training) had negative correlations with behavior. In addition, the regression analyses showed that only knowledge on color coded bins was the only external factor significant in predicting health workers' HCW segregation behaviors. The findings concerning the influence of external factors on HCW segregation behaviors suggest a need for further exploration of their roles related to HCW segregation behaviors.

### 4.4. Study Limitations

The major limitation to the study was that a self-administered questionnaire that relied on self-reported behavior by health workers was used. Most health workers reported that they segregated HCW. This is in contrast to the literature examined and some studies that employed direct observations. The use of the variable which noted segregation behavior for the last client served gave a better indication of this behavior. Self-reported surveys are reported to have the tendency to lead to overreporting of socially valued behavior since socially desirable responding is likely to occur in response to socially sensitive questions [34].

Indeed, this study might have overestimated the HCW segregation behavior of health workers through social desirability response bias where respondents tend to report positive behaviors more frequently than negative behavior. Therefore, for this study, the TPB was a predictor of self-perceptions of behavior rather than objective behavior [35].

## 5. Conclusion

Generally, health workers' HCW segregation behavior was high. However as shown by health workers' HCW segregation for the most recent client, a good number of health workers did not segregate HCW and therefore more needs to be done to further improve HCW segregation. The TPB significantly predicts health workers' HCW segregation behaviors although studies using self-reported data should be validated by additional objective measures. Interventions which aim at strengthening of health workers' perceived behavioral control and intentions could be effective in improving health workers' HCW segregation behaviors. Therefore, the district public health department should arrange educational seminars aimed at strengthening health workers' perceived behavioral control as well as intentions. Since health workers who had knowledge on the use of color coded reported that they did not segregate HCW, facility managers should go beyond improving knowledge to supporting practices by providing the resources required for HCW segregation, particularly the color coded bins.

## Figures and Tables

**Table 1:** Participants' characteristics.

Characteristic	*N*	%
*Sociodemographics*		
Gender		
Male	85	52.1
Female	78	47.9
*Professional characteristics*		
Type of training		
Doctor	4	2.5
Lab technician	24	17.2
Nursing assistant	32	19.6
Clinical officer	18	11.0
Nurse/midwife	83	50.9
Others	2	1.3
*Organizational characteristics*		
Level of HF		
HC II	39	23.9
HC III	74	45.4
HC IV	12	7.4
Hospital	38	23.3
Ownership of the HF		
Government	95	58.3
NGOs	68	41.7
*Personal characteristics *		
Perceived risk		
Yes	130	78.9
No	33	20.2
Knowledge on HCW segregation		
Yes	117	71.8
No	46	28.2
Knowledge on color coded bins		
Yes	39	23.9
No	124	76.1
Adequate training		
Yes	64	39.3
No	99	60.7
Department worked in		
OPD	44	27.0
Laboratory	26	16.0
Immunization	6	3.7
Wards	43	26.4
Others	44	27.0

HCF: Health Care Facility, HC: Health Center, NGO: Nongovernment Organization, OPD: Outpatient Department, HC II includes clinics.

**(a):** 

Variable	Intention	Perceived control	Subjective norms	Moral obligation	Attitude	Behavior
Intention	**1**	**.180** ^**∗**^	**.226** ^*∗∗*^	**.157** ^**∗**^	**.213** ^*∗∗*^	**.524** ^*∗∗*^
Perceived control		1	.494^*∗∗*^	.192^**∗**^	.246^*∗∗*^	**.452** ^*∗∗*^
Subjective norms			1	.061	.482 ^*∗∗*^	**.377** ^*∗∗*^
Moral obligation				1	.163 ^**∗**^	**.188** ^*∗∗*^
Attitude					1	**.293** ^*∗∗*^
Behavior						**1**

^**∗**^Correlation is significant at the 0.05 level (1-tailed). ^*∗∗*^Correlation is significant at the 0.01 level (1-tailed).

**(b):** 

Variable	1	2	3	4	5	6	7	8
(1) Ownership	1	−.051	−.061	.131	.268^*∗∗*^	.012	−.110	−.210^*∗∗*^
(2) Level of HF		1	−.163^**∗**^	.015	−.075	.002	−.149	.208^*∗∗*^
(3) Knowledge			1	.091	.266^*∗∗*^	.013	.281^*∗∗*^	−.299^*∗∗*^
(4) Risks				1	.108	.116	.092	−.228^*∗∗*^
(5) Knowledge on color coded bins						1	−.151	−.209^*∗∗*^
(6) Adequate training on segregation							1	−.176^**∗**^
(7) Behavior								1

^**∗**^Correlation is significant at the 0.05 level (1-tailed). ^*∗∗*^Correlation is significant at the 0.01 level (1-tailed).

**Table 3:** Regression analyses of behavior on attitudes, subjective norms, perceived control, and intention.

Variables	Unstandardized regression coefficient	Standard Error	Odds Ratio	*P* value
Attitude	1.96	1.74	7.08	.260
Subjective norms	.614	.85	1.85	.467
Perceived control	2.26	.61	9.61	<.001^*∗*^
Intention	2.90	.58	18.10	<.001^*∗*^

Nagelkerke *R* square = 0.525, Hosmer and Lemeshow *P* value = 0.958 (Hosmer and Lemeshow goodness-of-fit test indicates a good fit if *P* value is above 0.05), and ^*∗*^significant findings *P* value < 0.05.

**Table 4:** Multivariable regression analysis of TPB constructs adjusting for external variables to predict HCW segregation behavior.

Variables	Unstandardized regression coefficient	Standard Error	Odds Ratio	*P* value
Perceived behavioral control	2.647	.735	14.115	.000^*∗*^
Intention	2.836	.752	17.050	.000^*∗*^
Knowledge on color coded bins	−2.251	.705	.105	.001^*∗*^

Nagelkerke *R* square = 0.667, Hosmer and Lemeshow *P* value = 0.958 (Hosmer and Lemeshow goodness-of-fit test indicates a good-fit if *P* value is above 0.05) and ^*∗*^significant findings *P* value < 0.05.
